# Supremely elastic gel polymer electrolyte enables a reliable electrode structure for silicon-based anodes

**DOI:** 10.1038/s41467-019-13434-5

**Published:** 2019-12-06

**Authors:** Qingquan Huang, Jiangxuan Song, Yue Gao, Daiwei Wang, Shuai Liu, Shufu Peng, Courtney Usher, Alan Goliaszewski, Donghai Wang

**Affiliations:** 1 Department of Mechanical Engineering, The Pennsylvania State University, University Park, PA 16802 USA; 2 State Key Laboratory for Mechanical Behavior of Materials, Xi’an Jiaotong University, Xi’an, 710049 China; 3 Department of Chemistry, The Pennsylvania State University, University Park, PA 16802 USA; 4 Ashland Specialty Ingredients, Wilmington, DE 19808 USA

**Keywords:** Batteries, Batteries

## Abstract

Silicon-based materials are promising anodes for next-generation lithium-ion batteries, owing to their high specific capacities. However, the huge volume expansion and shrinkage during cycling result in severe displacement of silicon particles and structural collapse of electrodes. Here we report the use of a supremely elastic gel polymer electrolyte to address this problem and realize long-term stable cycling of silicon monoxide anodes. The high elasticity of the gel polymer electrolyte is attributed to the use of a unique copolymer consisting of a soft ether domain and a hard cyclic ring domain. Consequently, the displacement of silicon monoxide particles and volume expansion of the electrode were effectively reduced, and the damage caused by electrode cracking is alleviated. A SiO|LiNi_0.5_Co_0.2_Mn_0.3_O_2_ cell shows 70.0% capacity retention in 350 cycles with a commercial-level reversible capacity of 3.0 mAh cm^−2^ and an average Coulombic efficiency of 99.9%.

## Abstract (web summary)

Silicon-based anodes must maintain the electrode structure to fulfil their potential. Here the authors report the use of a supremely elastic gel polymer electrolyte to stabilize such anodes at electrode and particle levels, enabling superior battery performance with a commercial-level capacity.

## Introduction

**Fig. 1:**
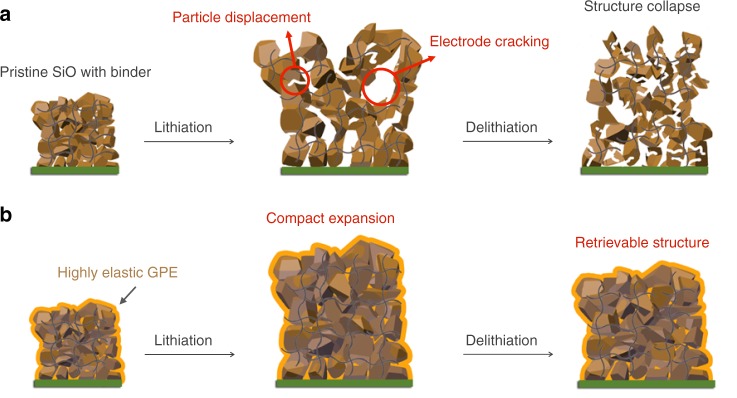
Schematic illustration of an elastic gel polymer electrolyte (GPE)-incorporated SiO anode with a reliable electrode structure. **a** Severe structural collapse of a conventional SiO electrode during lithiation and delithiation. The huge volume changes of SiO particles lead to the cracking at the particle level and electrode level. The blue lines represent the electrode binder. **b** The integrated structure of a SiO electrode during cycling enabled by a supremely elastic GPE, which serves as an intra-electrode cushion (orange) to reduce the thickness increase and cracking during lithiation and to help retrieve the electrode structure during delithiation.

Rechargeable lithium (Li)-ion batteries have been widely used in portable electronics, electric vehicles, and grid energy storage devices^1–3^. To increase the battery energy density, great efforts have been devoted to the development of high-capacity anode materials^4–7^. Silicon (Si)-based materials are extremely promising owing to their high specific capacities and low operation potentials^4,8–16^. Unfortunately, the huge volume changes of Si-based materials result in remarkable displacement of material particles and thickness increase of electrodes in the lithiation (Fig. 1a). This structural damage is not retrievable in the delithiation process, causing the loss of electrode integrity, unstable solid-electrolyte interphase (SEI) layers, and electrode peeling-off from current collectors^17–19^. The electrode correspondingly shows severe cracking in this process^6,20–23^. Consequently, the capacity fades quite rapidly, and this problem has become a major obstacle hindering the Si-based anode technology.

Despite the development of various structured Si-based materials^10,12,15,24–26^ and Si-carbon composites^27–32^, the displacement of material particles and structural collapse at the electrode level remains unsolved, especially when the mass loading of active materials is sufficiently high. To address this problem, different functional binders show the capability of alleviating the particle pulverization and maintaining the electrode integrity^24,33–41^. Besides, researchers have recently reported a polyrotaxanes-based polymer^42^ and self-healing polymers^43–46^ that can repair the damage and cracks in the electrode and maintain the integrity of micro-sized Si anodes.

In addition to the repair of electrode structure after cracking occurs, strategies to intrinsically alleviating the displacement of material particles and electrode cracking are highly needed. To this end, we here report a strategy using a supremely elastic gel polymer electrolyte (GPE), serving as an intra-electrode cushion to reduce the electrode expansion during lithiation and to facilitate the electrode to recover its structure during delithiation. We demonstrate a copolymer, namely poly(poly(tetramethylen-e ether) glycol-*co*-4,4’-methylene diphenyl diisocyanate)-ethylene diamine), enabling reliable electrode structure and stable cycling of micro-sized silicon monoxide (SiO) anodes at high loading conditions. The polymer is uniformly filled in the SiO electrode (Fig. 1b). It contains a soft ether-based domain and a hard cyclic ring-based domain, therefore, presenting excellent elasticity. Meanwhile, the addition of polymers within the electrode has generally deteriorated the Li ion transport. This polymer can uptake liquid electrolyte to form a GPE with an excellent ionic conductivity. Mechanical and morphological studies show that the use of the elastic GPE effectively reduces the absolute displacement of SiO particles and stabilizes electrode structure upon cycling. The severe electrode cracking and the peeling-off of SiO particles from the current collector are suppressed. SiO anodes with the elastic GPE have a reversible specific capacity of 1068 mAh g^−1^ in 250 cycles. When paring with a LiNi_0.5_Co_0.2_Mn_0.3_O_2_ (NCM) cathode, the cell displays capacity retention of 70.0% in 350 cycles with a reversible capacity of 3.0 mAh cm^−2^ and an average Coulombic efficiency of 99.9%.

## Results

### Chemical and physical properties of the elastic GPE

**Fig. 2:**
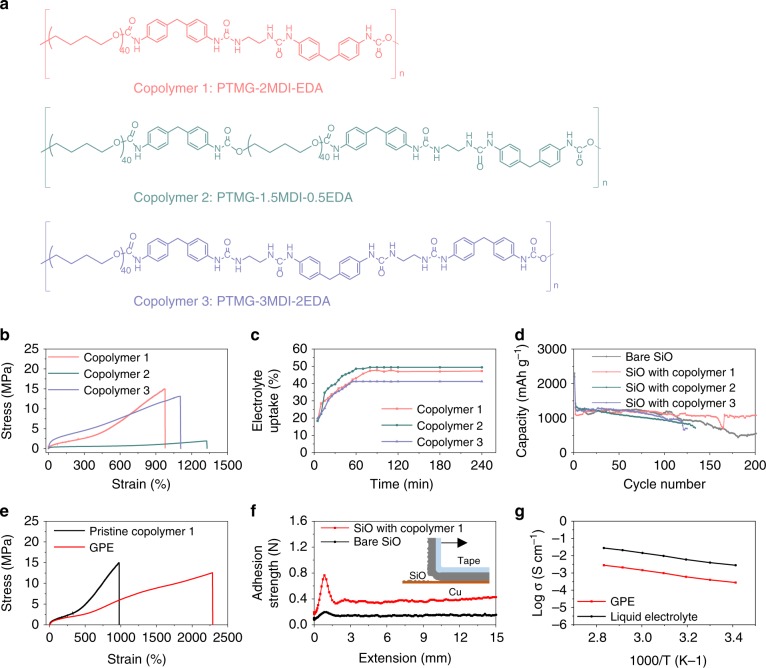
Structural optimization and characterization of the elastic GPE. **a** Structure of the copolymers, containing a soft domain (PTMG) and a hard domain (MDI-EDA) with different ratios. **b** Stress-strain curves of the copolymers. **c** Swelling test of the polymer membranes in a 1 M LiPF_6_ in EC/DEC electrolyte. **d** Cycling stability of the SiO electrodes with the copolymers. The mass loading of SiO electrodes is 3.7 mg cm^−2^, and the copolymer amount is 0.4 mg cm^−2^. **e** Stress-strain curves of the copolymer 1 before and after swelling. **f** Peel adhesion of SiO electrodes. The electrode was peeled-off from the Cu current collector using a 90-degree dragging force. **g** Ionic conductivity measurement of the elastic GPE and liquid electrolyte as a function of temperature.

We utilized a soft ether-based domain, poly(tetramethylene ether) glycol (PTMG), and a hard cyclic ring-based domain, 4,4’-methylene diphenyl diisocyanate)-ethylenediamine (MDI-EDA), to compose the copolymer for GPE (Supplementary Figs. 1–3). The soft domain can swell in carbonate electrolytes; and the hard domain can prevent excessive swelling of the entire copolymer and has chemical weak interactions (hydrogen bonding and π–π interactions) within the polymer chains. This unique soft-hard combined structure not only provides the copolymer with supreme elasticity but also helps maintain the electrode integrity with cycling. We synthesized three copolymers with different ratios of the soft and hard domains (Fig. 2a) and studied their chemical and physical properties used as GPE.

To compare the performance of the copolymers, we examined the mechanical strength and electrolyte swelling of the copolymers, and cycle life of the SiO electrodes incorporating the copolymers as GPE. The copolymer 1 with a proper MDI-EDA (hard)-PTMG (soft) ratio of 2:1 presents the optimal elongation capability, electrolyte swelling amount, and stabilization of SiO anodes. Figure 2b shows the rubbery property measured by a stress-strain test. The copolymer 1 can tolerate an elongation of above 900% and presents a modulus of 1.2 MPa. Meanwhile, it swelled in a carbonate electrolyte, 1 M lithium hexafluorophosphate (LiPF_6_) in ethylene carbonate (EC)/diethyl carbonate (DEC) (1:1 v/v) electrolyte with up to 48 wt.% electrolyte uptake (Fig. 2c and Supplementary Fig. 4). In comparison, the copolymer 2, having less MDI-EDA hard domains, exhibits a low modulus of 0.5 MPa with up to 50 wt.% electrolyte uptake; and the copolymer 3, containing more MDI-EDA hard domains, has a high modulus of 2.4 MPa but poorly swelled in the electrolyte. Correspondingly, the SiO anode incorporating the copolymer 1 as GPE shows the optimal cycling stability in the Li|SiO half cells. As shown in Fig. 2d, the SiO electrode with the copolymer 1 had a stable capacity of 1068 mAh g^−1^ in 150 cycles. After replacing with a fresh Li counter electrode^42^, the cell capacity arose to 1068 mAh g^−1^ and remained stable over 250 cycles. In contrast, the capacities of the cells incorporating the copolymers 2 and 3 faded after 30 and 70 cycles, which is similar to the bare SiO anode (Fig. 2d). Together, the copolymer 1 shows good mechanical strength and electrolyte swelling and the formed GPE enables the optimal stabilization of SiO electrodes.

We further characterized the mechanical properties of the optimal elastic GPE. The GPE showed even better mechanical property than the dry copolymer 1. It tolerated more than 2300% elongation upon stretching before breakdown (Fig. 2e), which is more than twice larger than the copolymer 1. The Young’s modulus is 0.7 MPa, indicating that the GPE remains highly elastic. At the electrode level, the GPE cushion is evenly distributed within the electrode. The as-prepared SiO electrode has a smooth surface (Supplementary Figs. 5, 6). X-ray photoelectron spectroscopy (XPS) analysis indicates a uniform covering of the polymer on the SiO surface, evidenced by the observation of N signals (Supplementary Fig. 7)^47^. Meanwhile, we found uniformly distributed N signals throughout the electrode in the cross-sectional energy dispersive X-ray spectrometry (EDS) images (Supplementary Fig. 8). With the elastic GPE, strong adhesion between SiO particles and current collectors was realized, as evidenced by a peel-adhesion test (Fig. 2f). The uncycled SiO electrode with the copolymer has an adhesion strength of 0.4 N, while that of the bare SiO electrode is only 0.2 N. Furthermore, after vigorously stirring the SiO electrodes in a solvent, we found that the control SiO electrode was seriously damaged as a large amount of SiO particles were peeled off (Supplementary Fig. 9). Surprisingly, the SiO electrode with the copolymer is quite stable, further indicating a strong adhesion. Besides, the elastic GPE displays an ionic conductivity of 2.4 × 10^−4^ S cm^−1^ at 25 °C, comparable to that of the liquid electrolyte (Fig. 2f and Supplementary Fig. 10). Moreover, cyclic voltammetry studies imply that the GPE is stable in a wide electrochemical window (up to 4.5 V versus Li^+^/Li) (Supplementary Fig. 11).

### Structural evolution of SiO electrodes upon cycling

**Fig. 3:**
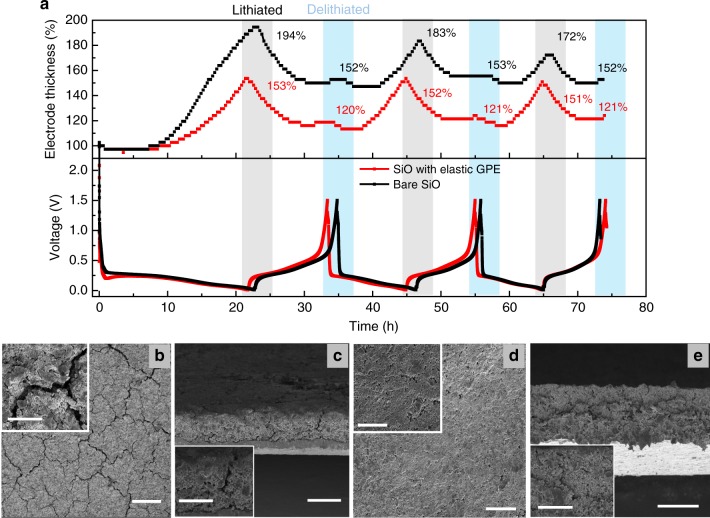
Characterization of the SiO electrode with an elastic GPE. **a** In situ measurement of thickness evolution of a control SiO electrode and a SiO electrode with the elastic GPE in the initial three lithiation/delithiation cycles at a current density of 0.3 mA cm^−2^ between 0.01 and 1.5 V. The mass loading of SiO electrodes is 3.7 mg cm^−2^ and the copolymer amount is 0.4 mg cm^−2^. **b**, **c** Top-view (**b**) and cross-sectional (**c**) SEM images of the control SiO electrode after 10 cycles. **d**, **e** Top-view (**d**) and cross-sectional (**e**) SEM images of the SiO electrode with the elastic GPE after 10 cycles. Scale bars: 100 μm (**b**, **d**), 20 μm (insets in **b**, **d**), 50 μm (**c**, **e**), 20 μm (insets in **c**, **e**).

To study the integrity of SiO electrodes upon cycling, we in situ measured the changes in electrode thickness during repeated lithiation and delithiation processes. The initial thicknesses of the electrodes were normalized as 100%. At the beginning of the initial lithiation process, the SiO electrodes with and without the elastic GPE have no obvious changes in the electrode thickness. This is because the electrodes are porous and can accommodate the expansion of SiO particles. Upon lithiation, the electrode thickness began to increase rapidly. The control SiO electrode shows a large thickness increase of 94% (Fig. 3a). Surprisingly, the thickness increase of the SiO electrode with the elastic GPE is only 53%, which is significantly reduced compared to the conventional SiO electrode. In the following delithiation, the electrodes correspondingly shrank. The thickness of the SiO electrode with the elastic GPE was dropped to 120%. The 20% of thickness increase of the electrode can be assigned to the formation of the SEI layer, inactive Li_2_O, and lithium silicate. Contrastingly, the control electrode has a 52% thickness increase at the delithiation status, resulting from severe cracking at the electrode level. The SiO electrode with the elastic GPE maintains stable thickness changes in the following cycles, indicating a reliable electrode structure without severe cracking and damage.

We observed the electrode morphology using scanning electron microscopy (SEM) to further investigate the structural integrity of the SiO electrodes. The control SiO electrode presents large cracks only after 10 cycles (Fig. 3b). The cross-sectional SEM image showed large cracks within the electrode, which causes electrode delaminating from current collectors (Fig. 3c). The cracks continued to grow after 20 cycles (Supplementary Fig. 12). In contrast, the SiO electrode with the elastic GPE had much fewer cracks after 10 cycles (Fig. 3d). The cross-sectional SEM image displayed minor cracks and the electrode is very dense (Fig. 3e). This morphology remained after 20 cycles (Supplementary Fig. 12), indicating good reliability of the electrode structure. We also studied the impedance evolution of Li|SiO cells with cycling, the cell incorporating the elastic GPE had stable resistances without a clear increase in 100 cycles, while the resistances of the control sample grew rapidly (Supplementary Fig. 13). Together, all these findings verify the dramatically improved electrode integrity of the SiO electrode during repeated lithiation and delithiation processes.

### Interfacial stability of SiO anodes

**Fig. 4:**
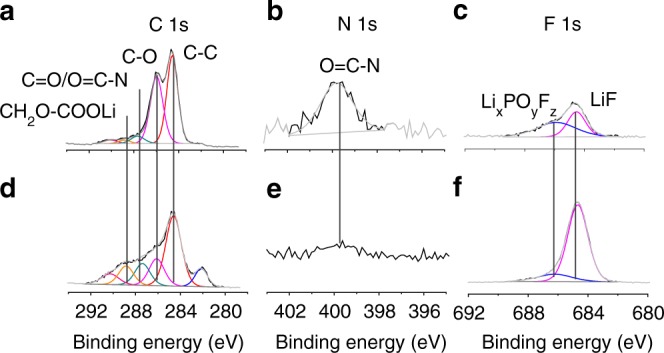
High-resolution XPS spectra of cycled SiO electrodes. **a**–**c** C 1s (**a**), N 1s (**b**), and F 1s (**c**) spectra of a SiO electrode with the elastic GPE. **d**–**f** C 1s, (**d**) N 1s (**e**), and F 1s (**f**) spectra of a control SiO electrode.

We next investigated the SEI stability by conducting high-resolution XPS on the surface of SiO electrodes after 10 cycles. In the XPS spectra of a cycled SiO electrode with the GPE, characteristic O=C-N signals (peaks at 399.7 eV in the N 1s spectrum and 287.6 eV (overlapped with C=O) in the C 1s spectrum) of the copolymer 1 were observed (Fig. 4a, b)^46^. The concentration of inorganic Li salts in the SEI (Fig. 4c) is markedly lower than that in the control sample (Fig. 4f)^47^, which include Li_x_PO_y_F_z_ (the peak at 686.2 eV in the F 1s spectrum)^9^, LiF (the peak at 684.8 eV in the F 1s spectrum), Li_2_CO_2_R (the peak 287.7 eV in the C 1s spectrum), and CH_2_OCOOLi (the peak 288.9 eV in the C 1s spectrum). The concentration of organic components, which include C-C/C-H (the peak at 284.6 eV in the C 1s spectrum) and C-O (286.0 eV in the C 1s spectrum) at the surface of the SiO with the elastic GPE is higher than that in the SEI layer of the control SiO electrode (Fig. 4a, d). These results imply that the elastic GPE may participate in the SEI formation and thus reduce electrolyte decomposition.

### Electrochemical performance of SiO anodes

After confirming the electrode integrity, we evaluated the cycling stability of SiO anodes. We optimized the copolymer amount in the electrode. The GPE-incorporated SiO anode had a decreased specific capacity when excessive copolymer was used (Supplementary Fig. 14). The SiO anode with a copolymer amount of 0.4 mg cm^−2^ displayed a decent capacity and rate capability (Supplementary Fig. 15). We also compared the elastic GPE with other polymers used as the cushion, including polyacrylic acid (PAA), polyvinyl alcohol (PVA), PAA-PVA, and poly(vinylidenefluoride-hexafluoropropylene) (PVdF-HFP). These polymers presented poor elasticity and ionic conductivity, resulting in reduced cycling stability and large polarization of SiO anodes (Supplementary Fig. 16). It is also noted that to use the copolymer as additional binders showed no improvements on the SiO anode stability (Supplementary Fig. 17). These findings indicate the importance of using a highly elastic GPE as the cushion.

**Fig. 5:**
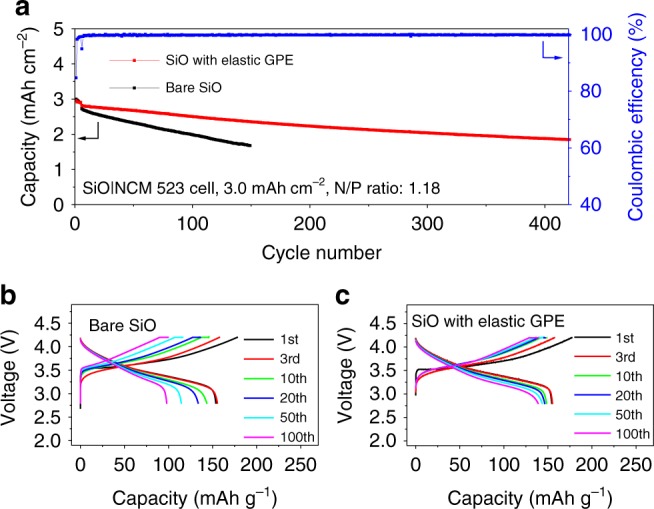
Cycling stability of SiO|NCM cells. **a** Discharge capacities of SiO|NCM 523 cells at a current density of 1.0 mA cm^−2^ and the Coulombic efficiency of the cell incorporating the elastic GPE. **b**, **c** Voltage profiles of the SiO|NCM 523 cells with (**b**) and without (**c**) the elastic GPE. Pre-cycled SiO electrodes were used for full-cell assembling. The mass loading of SiO anodes is 4.7 mg cm^−2^, and the copolymer amount is 0.4 mg cm^−2^.

We next tested the cycling stability of SiO|NCM full cells. To compensate for the irreversible loss of active Li, the SiO anodes were pre-cycled for 1 cycle and displayed a reversible capacity of 4.0 mAh cm^−2^. Then the SiO anodes were paired with fresh NCM cathodes (3.4 mAh cm^−2^. The cell incorporating the elastic GPE had an initial charge capacity of 3.5 mAh cm^−2^ and an initial discharge capacity of 3.0 mAh cm^−2^. A capacity retention of 70.0% in 350 cycles was achieved (Fig. 5a), associated with limited cell polarization (Fig. 5b). In contrast, the control cell presented capacity retention of merely 70.0% in 76 cycles and had severe polarization (Fig. 5c). We calculated the Coulombic efficiency-cycle life relationship (Supplementary Fig. 18) and found that it is pivotal to reach a Coulombic efficiency of 99.9% for Si-based anodes in order to realize a capacity retention of 80% in 225 cycles, which is promising for practical use^48,49^. The use of the highly elastic GPE enabled a Coulombic efficiency of 99.9% in 300 cycles. Note that the use of the elastic GPE stabilized the micro-sized Si electrode as well (Supplementary Fig. 19).

## Discussion

In this work, we report a supremely elastic gel polymer electrolyte that intrinsically alleviates the damage and cracking of Si-based anodes at the electrode level. This involves the use of a unique copolymer containing soft and hard domains. The increase of electrode thickness and the displacement of SiO particles were effectively reduced, realizing a reliable electrode structure upon cycling. Significantly improved cycle life and efficiency of SiO|NCM 523 cells were achieved at high-capacity conditions. Using the approach demonstrated in this work, more functional GPEs can be designed for practical high-performance Si-based anodes.

## Methods

### Preparation of the copolymer

Firstly, MDI (4,4’-methylene diphenyl diisocyanate, Sigma-Aldrich) (2.0 mmol) and PTMG (poly(tetramethylene ether) glycol, Mw = 2900, Sigma-Aldrich) (1.0 mmol) were added in an anhydrous dimethylacetamide (Sigma-Aldrich) solution (20 ml), and the mixture was heated at 80 °C for 4 h under inert argon gas to form isocyanate capped PTMG intermediate. After cooling to room temperature, EDA (ethylenediamine, Sigma-Aldrich) (1.0 mmol) was added to the solution under vigorous stirring, and the mixture was heated to 70 °C for 4 h. The polymer can be obtained by evaporating the solvent at 60 °C.

### Preparation of SiO anode and NCM cathode

To investigate the integrity of SiO anodes at an electrode level, we performed mechanical and morphological characterizations on SiO electrodes. The SiO electrodes were prepared by mixing SiO powder (~10 µm), conductive carbon (Timical), and polyacrylic acid-polyvinyl alcohol (PAA-PVA) binder^37^ with a mass ratio of 7:2:1 to form a slurry. The solid content of the slurry was ~30 wt%. The slurry was cast on a copper foil using a doctor blade. The electrode was dried at 100 °C for 2 h and 150 °C for 2 h under vacuum. The mass loadings of the SiO electrodes for the half and full cells are 3.7 mg cm^−2^ and 4.7 mg cm^−2^, respectively. The SiO electrodes with the copolymer were prepared by a solution cast coating method. The polymer solution (5 wt.% in dimethylacetamide) was cast on a SiO electrode using a doctor blade, and then it can self-infuse into the electrode. The solvent was evaporated at room temperature overnight, and the electrode was further dried at 120 °C for 2 h under vacuum. For the SiO electrodes with the mass loadings of 3.7 and 4.7 mg cm^−2^, the amount of the copolymer used is 0.4 and 0.7 mg cm^−2^, respectively. NCM cathodes were prepared by mixing NCM 523 powders, conductive carbon (Timical), and polyvinylidene fluoride (PVDF) (Sigma-Aldrich) at a mass ratio of 90:5:5 in N-methyl-2-pyrrolidone (Sigma-Aldrich) to form slurry. The solid content of the slurry was ~45 wt%. After casting the solution on an aluminum foil, the electrode was dried at 100 °C under vacuum for 12 h. The mass loading of NCM 523 active material was 20 mg cm^−2^.

### Electrochemical tests

Half-cell tests were performed in a CR 2016 type coin cell assembled with Li metal (250 µm) as the counter electrode. The electrolyte was 1 M LiPF_6_ in EC/DEC (v/v 1:1, BASF) with 10 w% fluoroethylene carbonate (FEC, BASF) additive. Galvanostatic cycling test was carried out at 0.3 mA cm^−2^ for the first cycle and 1.0 mA cm^−2^ for the rest cycles between 0.01 V and 1.5 V on a BT2000 battery testing system (Arbin Instruments, USA). The specific capacity was calculated on the SiO active material. For full-cell tests, the SiO anodes were pre-charged and pre-discharged for one cycle with a current density of 0.3 mA cm^−2^. The pre-cycled SiO electrodes with a mass loading of 4.7 mg cm^−2^ and a reversible capacity of 4.0 mAh cm^−2^ were paired with NCM cathodes (mass loading of 20 mg cm^−2^, reversible capacity of 3.4 mAh cm^−2^) with an N/P ratio of 1.18. The full cell was cycled between 2.8 and 4.2 V at a small current density of 0.3 mA cm^−2^ for 3 cycles and then cycled at 1.0 mA cm^−2^ under a constant current-constant voltage mode. At the end of each charge cycle, the cell was held at 4.2 V until the current density drops below 0.3 mA cm^−2^. To conduct the swelling test, the polymer membrane was cut into a round disk (40 mg, 12 mm in diameter and 500 µm in thickness) and then immersed to a 1 M LiPF_6_ in EC/DEC (v/v 1:1, BASF) electrolyte in an argon-filled glovebox. The membrane weights were measured after various time periods. To measure the ionic conductivity, the saturated membrane was assembled in a coin cell with two stainless steel spacers as working and counter electrode, respectively. The ionic conductivity was measured as a function of temperature using electrochemical impedance spectroscopy (0.1 Hz–10^5^ Hz). For the cyclic voltammetry test, the polymer membrane was sandwiched between a stainless steel working electrode and a Li metal counter electrode in a coin cell. The cell was cycled between −0.2 and 4.6 V at a scan rate of 0.2 mV s^−1^.

### In situ electrode thickness method

The SiO anode electrode was assembled into an HS Swell Analysis Cell from Hoshen Corp in a glove box. After assembly, the test cell was removed from the glove box and placed in the test cell stand that holds a Mitutoyo ID-S digital micrometer. The test cell was connected to a channel on the Maccor battery tester 4000. The change in electrode thickness every thirty seconds was recorded while the test cell was charging and discharging.

### Characterizations

Fourier-transform infrared spectroscopy (FT-IR) was performed using a Bruker IFS 66/S FT-IR spectrometer and Spectra-Tech Collector II DRIFTS accessory. The electrochemical impedance and cyclic voltammetry were tested in Solartron 1260 equipment. X-ray photoelectron spectroscopy (XPS) was conducted with a Kratos Analytical Axis Ultra XPS using an air-sensitive holder. The surface morphologies and thickness of the SiO electrodes before and after cycling were investigated with a NanoSEM 630 scanning electron microscope. The electrode surface roughness was measured with atomic force microscopy (AFM) by the tip indentation technique. We measured the strain-stress behavior of the GPE membrane in EC/DEC (1:1 v/v) solution, because LiPF_6_ salt is sensitive to moisture.

## Supplementary information

Supplementary Information

## Data Availability

The datasets generated during and/or analyzed during the current study are available from the corresponding author on reasonable request.
