# Supplementary material for: Supremely elastic gel polymer electrolyte enables a reliable electrode structure for silicon-based anodes
Source: Nat Commun. 2019 Dec 6;10:5586. doi: 10.1038/s41467-019-13434-5 (PMC6898440; doi:10.1038/s41467-019-13434-5)
Supplement: Supplementary file 1 — Supplementary Information [file 41467_2019_13434_MOESM1_ESM.pdf]

Supplementary Information

Supremely elastic gel polymer electrolyte enables a reliable electrode structure for silicon-based anodes

Huang et al.

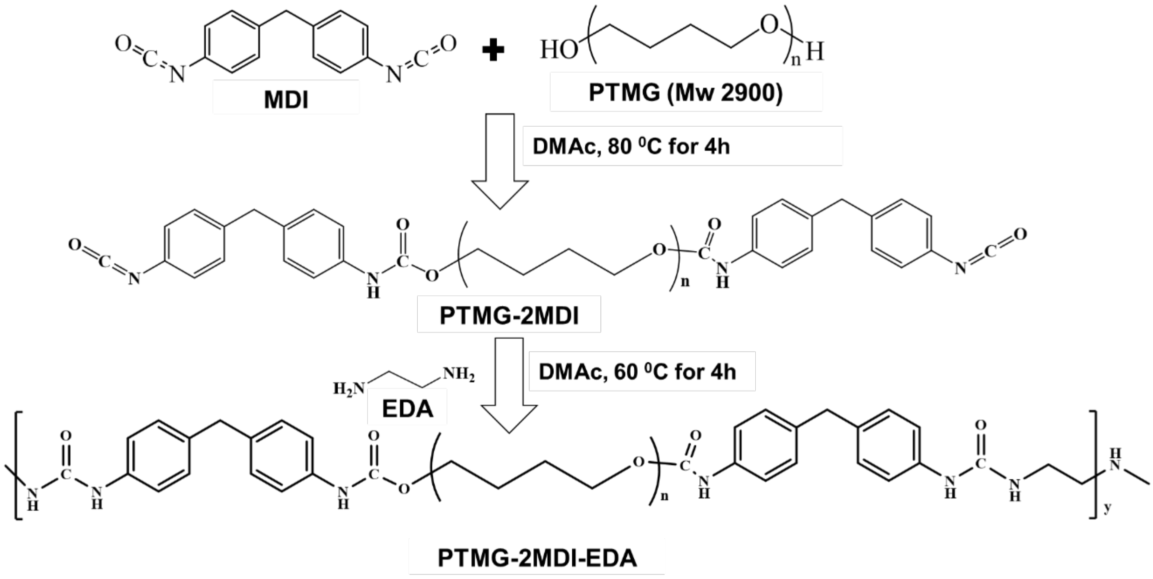

Supplementary Figure 1. Schema of synthesis of the copolymer 1.

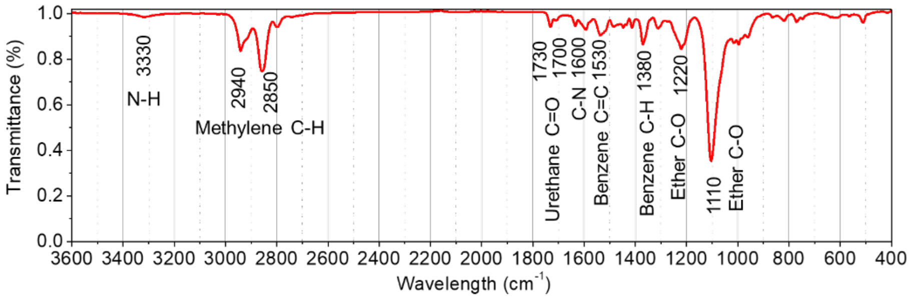

Supplementary Figure 2. Fourier transform infrared (FTIR) spectrum of the copolymer 1.

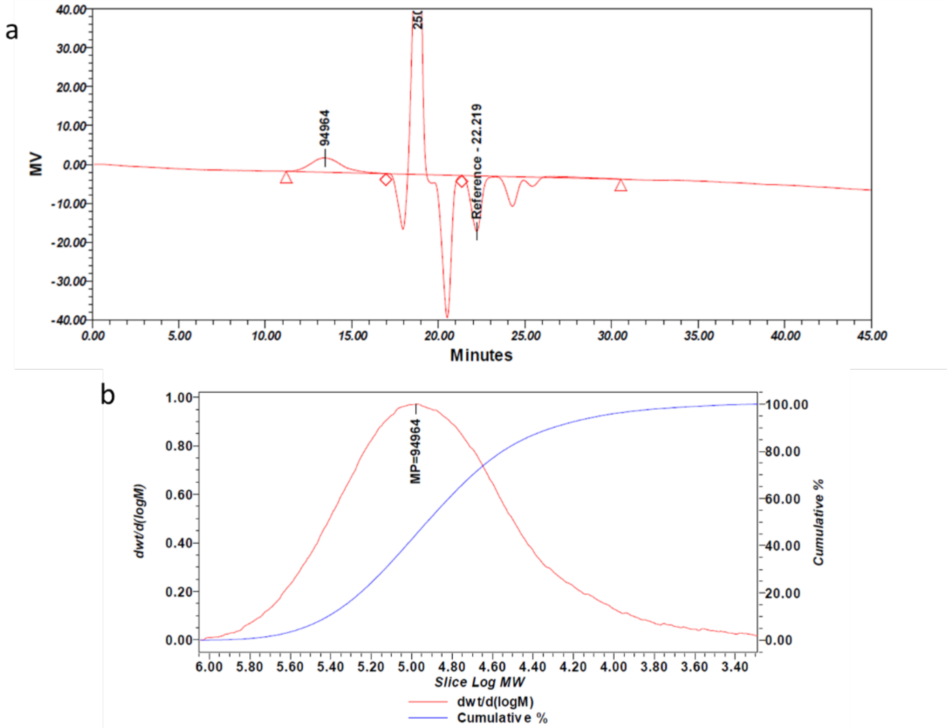

Supplementary Figure 3. Gel permeation chromatography (GPC) analysis of the copolymer 1. **(a)** Elution time of copolymer 1, **(b)** molecular weight distribution of copolymer 1. The copolymer 1 has a Mn of 40,000 g mol^{-1} and a Mw of 118,000 g mol^{-1}. The polymer showed high polydispersity index of 2.9.

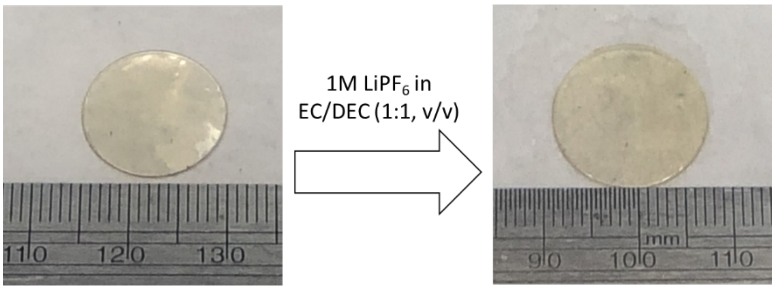

Supplementary Figure 4. Optical images of the copolymer 1 membrane swelling experiment in a liquid electrolyte. The dry copolymer membrane has a diameter of 15 mm and a thickness of 450 μm. After swelling in liquid electrolyte, the membrane diameter increased to 17 mm while its thickness was still around 450 μm. Thus the copolymer swelling process results in 28% volume increase.

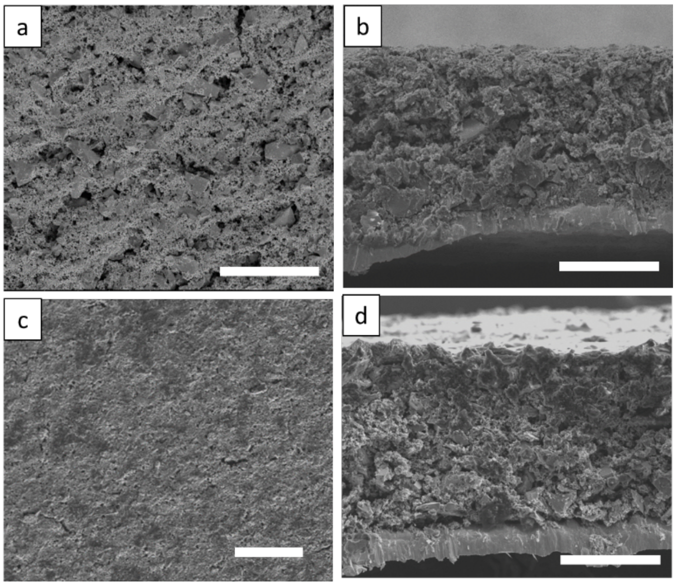

Supplementary Figure 5. SEM characterization of pristine SiO electrodes with different amounts of the copolymer 1. (**a**) Top-view and cross-section (**b**) of SiO electrode with 0.4 mg cm^{-2} polymer coating. (**c**) Top-view and cross-section (**d**) of SiO electrode with 0.7 mg cm^{-2} polymer coating. Scale bars, 30 µm (**a**-**d**).

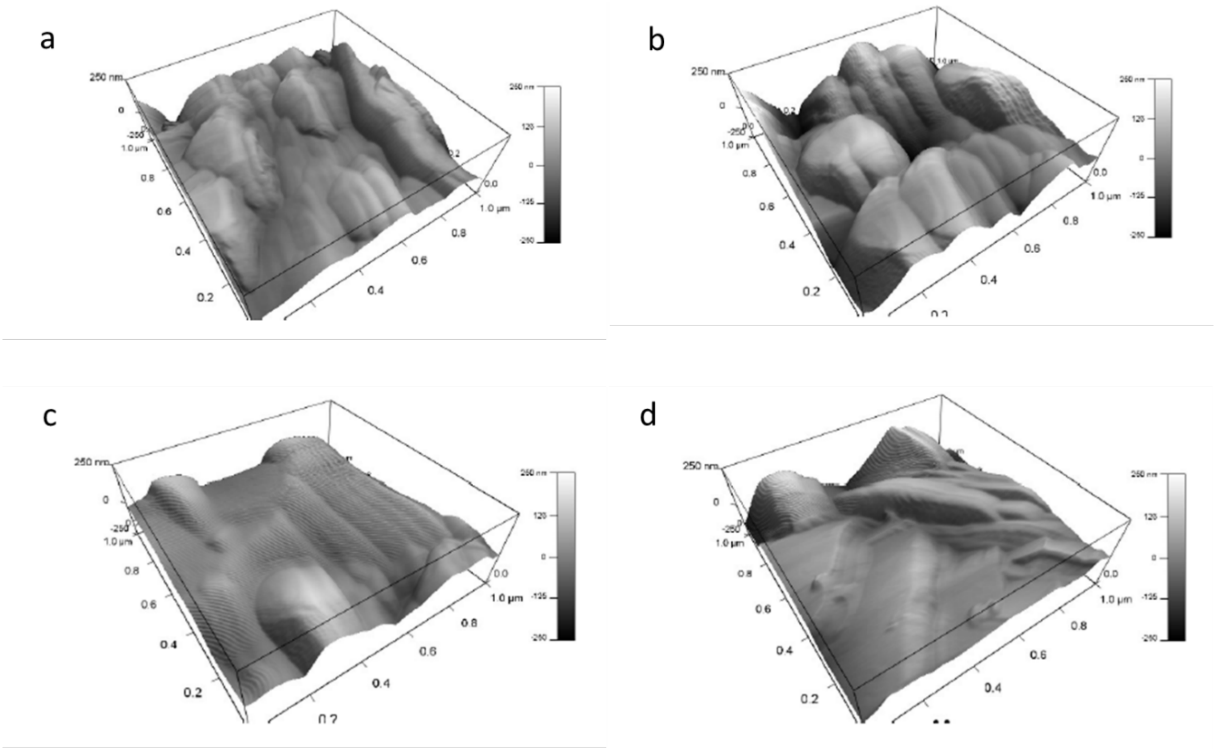

Supplementary Figure 6. Atomic force microscopy (AFM) characterization of morphology for SiO electrodes. **(a**, **b)** pristine bare SiO electrodes, **(c**, **d)** pristine SiO electrodes with the 0.4 mg cm^{-2} copolymer 1.

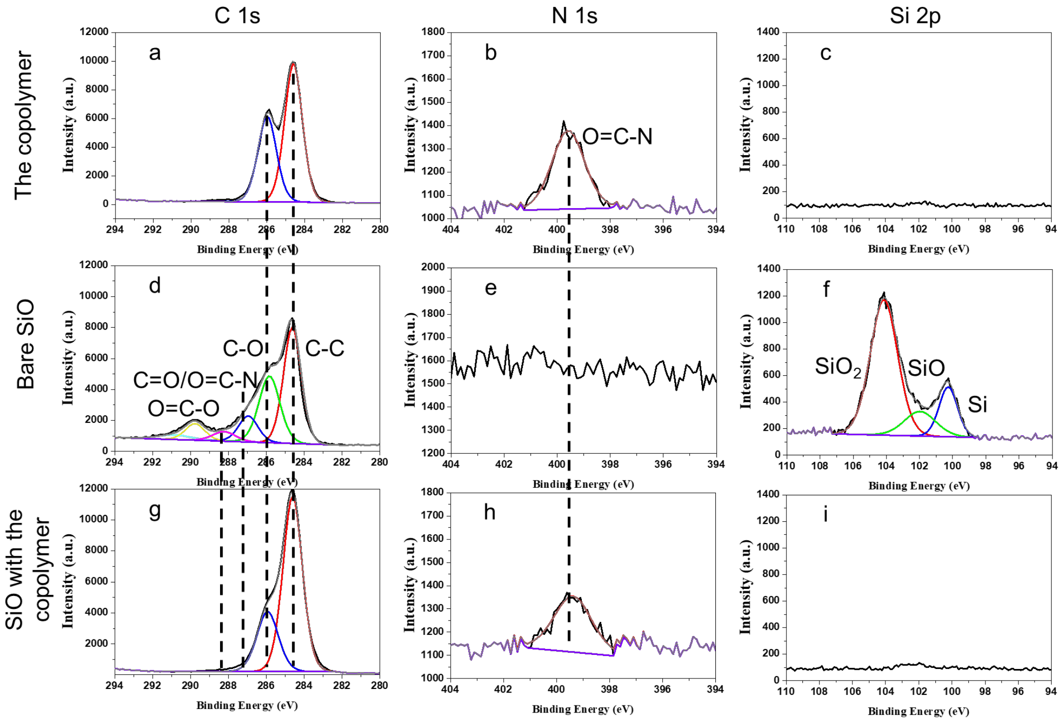

Supplementary Figure 7. High resolution XPS spectra of pristine SiO electrodes. (**a-c**) the copolymer 1 coated on Cu, (**d-f**) a bare SiO electrode, and (**g-i**) a SiO electrode with the copolymer 1 coating. (**a**) (**d**) and (**g**) are the C 1s spectra, (**b**) (**e**) and (**h**) are the N 1s spectra, (**c**) (**f**) and (**i**) are the C 1s spectra.

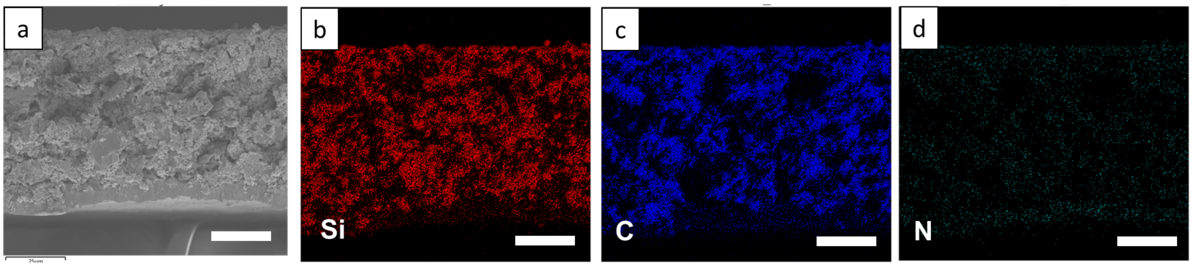

Supplementary Figure 8. Characterization of polymer distribution in SiO electrode. (**a**) Cross-sectional SEM image and (**b-d**) EDS mapping images of a pristine SiO electrode with the copolymer 1 for corresponding elements of (**b**) Si, (**c**) C, and (**d**) N. Scale bars, 25 µm (**a-d**).

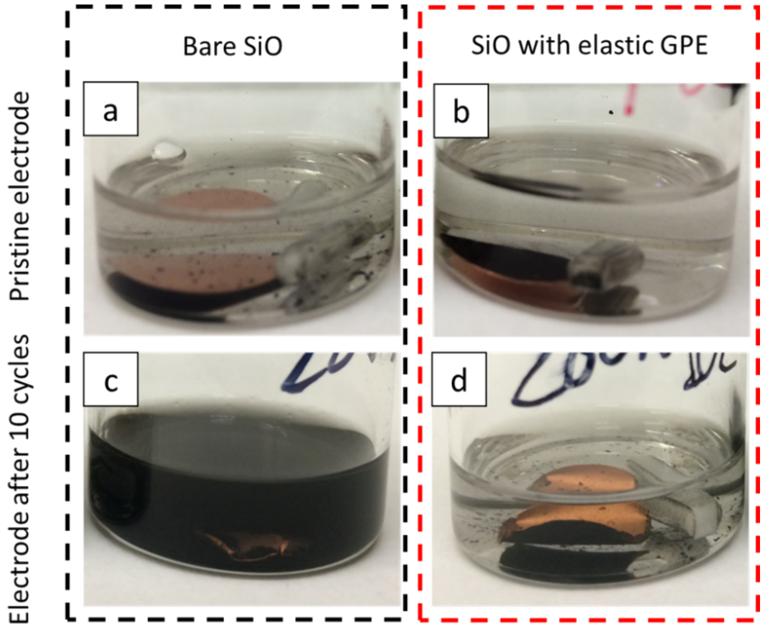

Supplementary Figure 9. Electrodes stability test in liquid electrolyte under magnetic stirring. (**a**) a pristine bare SiO electrode, (**b**) a pristine SiO electrode with the elastic GPE, (**c**) a bare SiO electrode after 10 cycles, (**d**) a SiO electrode with the elastic GPE after 10 cycles.

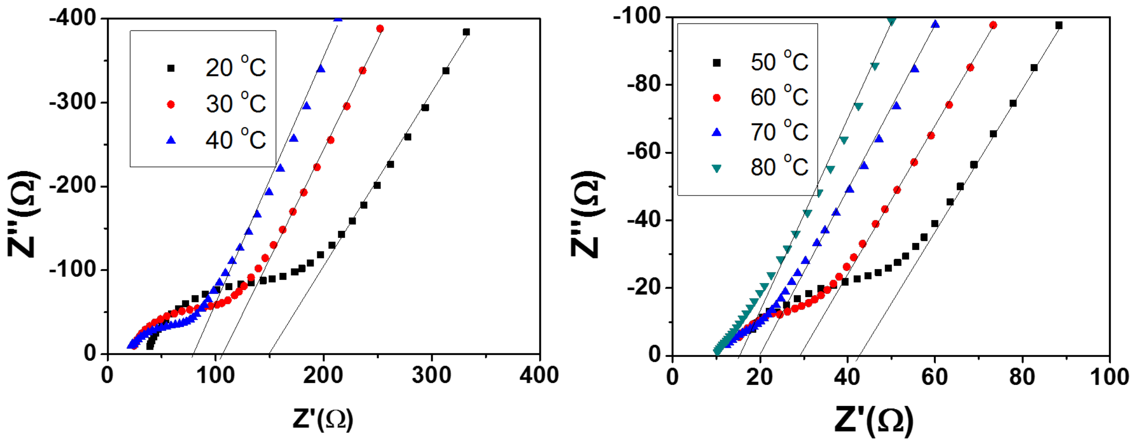

Supplementary Figure 10. Electrochemical impedance spectra (EIS) of the copolymer 1 GPE membrane at different temperatures. The membrane is 500 μm in thickness and 1.2 cm^2 in area.

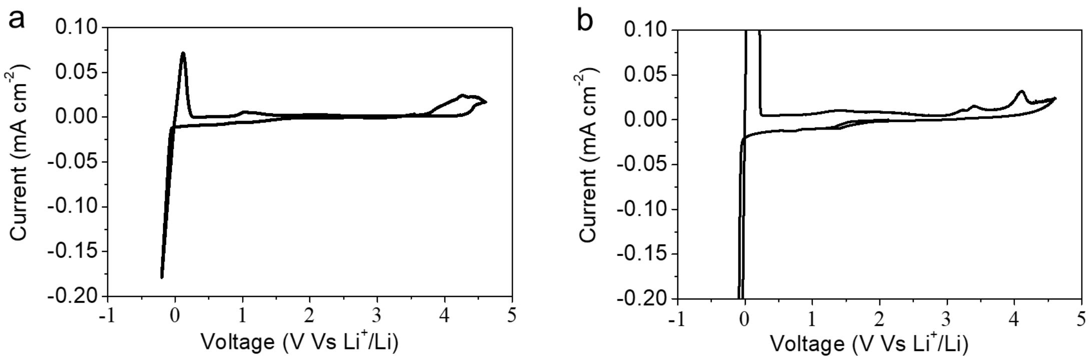

Supplementary Figure 11. Cyclic voltammetry test of (**a**) the copolymer 1 GPE and (**b**) liquid electrolyte (1 M $LiPF_6$ in EC/DEC).

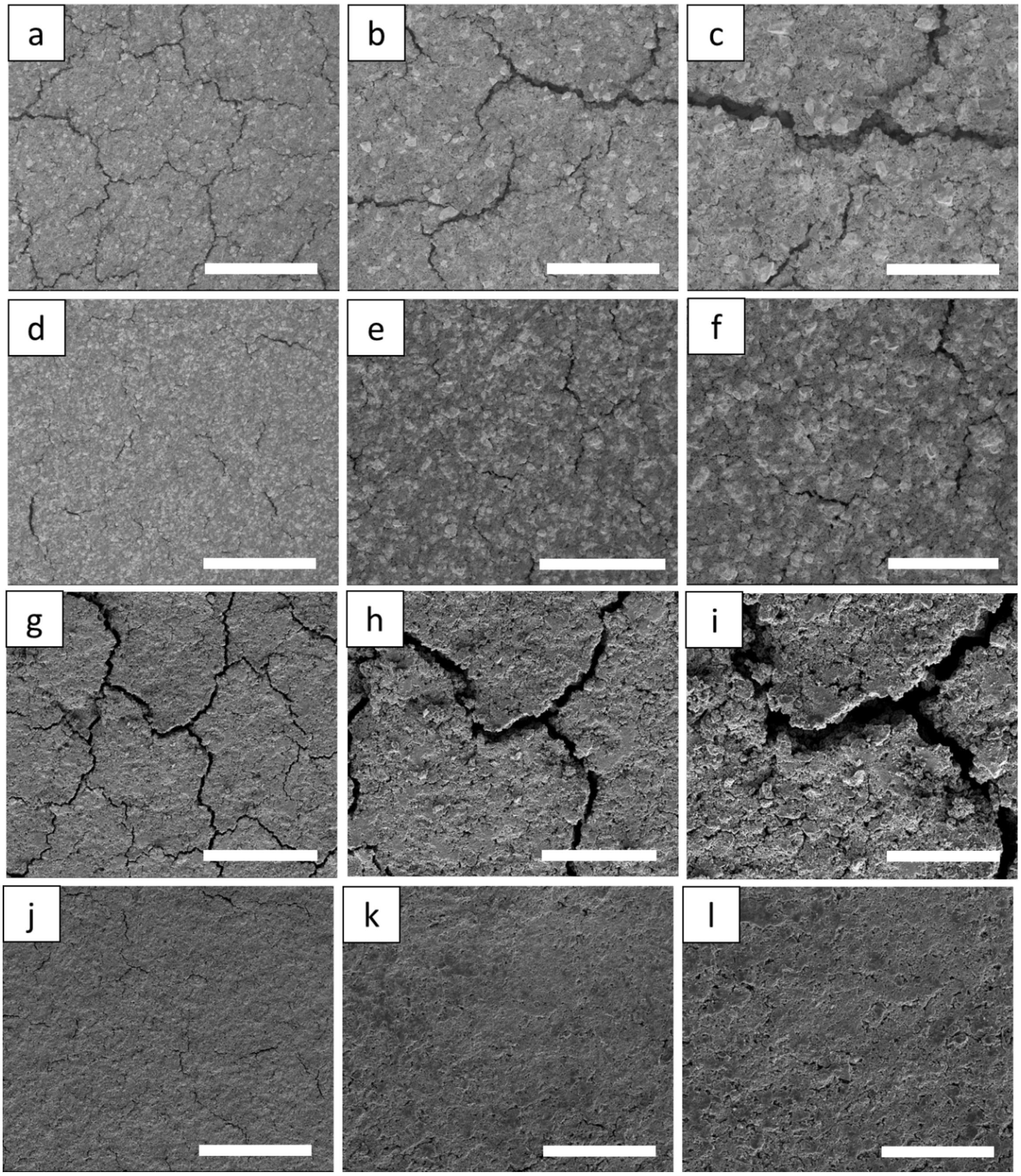

Supplementary Figure 12. Morphology characterization of bare SiO electrodes and SiO electrodes with the elastic GPE after different cycles. (**a-c**) SEM images of bare SiO electrodes after 1 cycle, (**d-f**) SEM images of SiO electrodes with the GPE after 1 cycle, (**g-i**) SEM images of bare SiO electrodes after 20 cycles, (**j-k**) SEM images of SiO electrodes with the elastic GPE after 20 cycles. Scale bars, 200 µm (**a**, **d**, **g**, **j**), 100 µm (**b**, **e**, **h**, **k**), 50 µm (**c**, **f**, **i**, **l**).

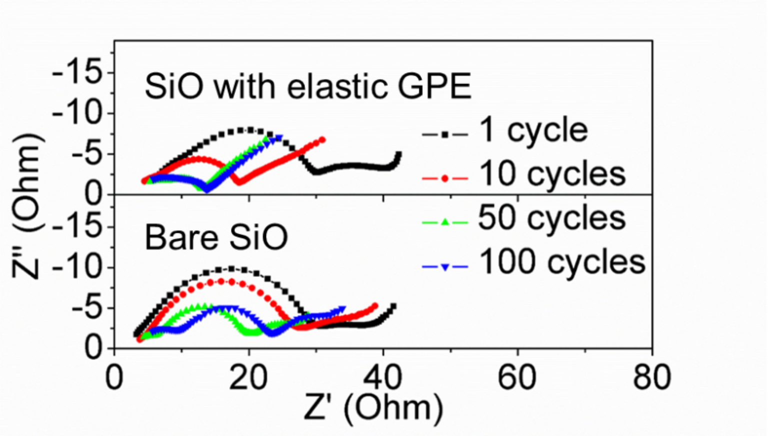

Supplementary Figure 13. Electrochemical impedance spectra (EIS) curves of Li|SiO cells after 1, 10, 50, and 100 cycles. The mass loading of SiO electrodes is 3.7 mg cm^{-2} and the polymer coating amount is 0.4 mg cm^{-2}.

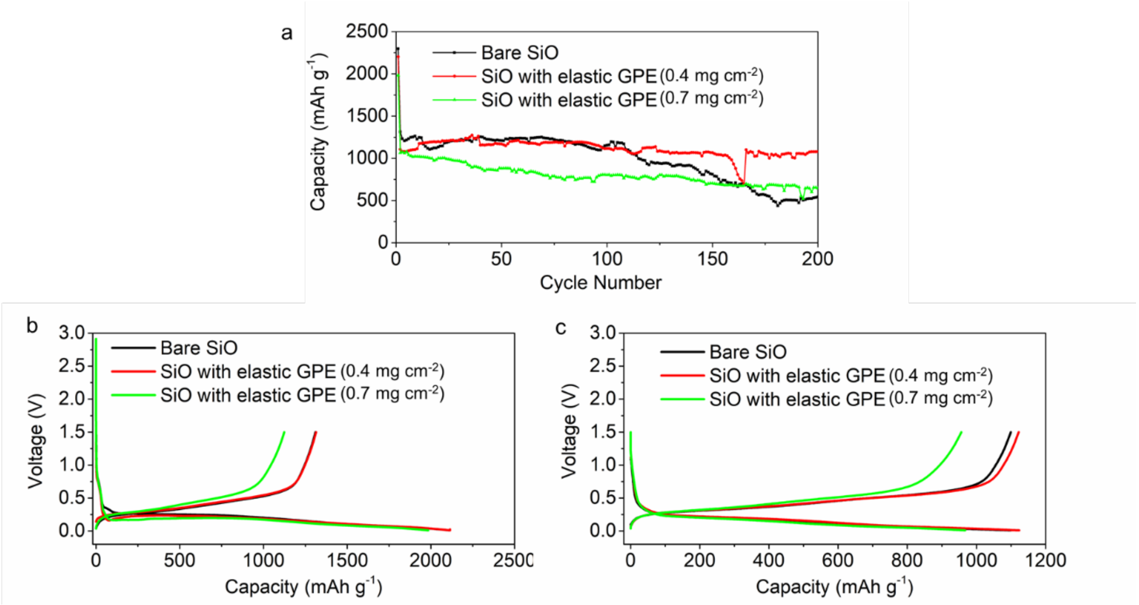

Supplementary Figure 14. Electrochemical performance of SiO electrode with different amounts of the copolymer 1. (a) Discharge capacity of SiO electrodes with different GPE coating amount. The mass loading of the SiO electrodes is 3.7 mg cm^{-2}. (b) the 1st cycle and (c) the 30th cycle voltage profile of SiO electrodes.

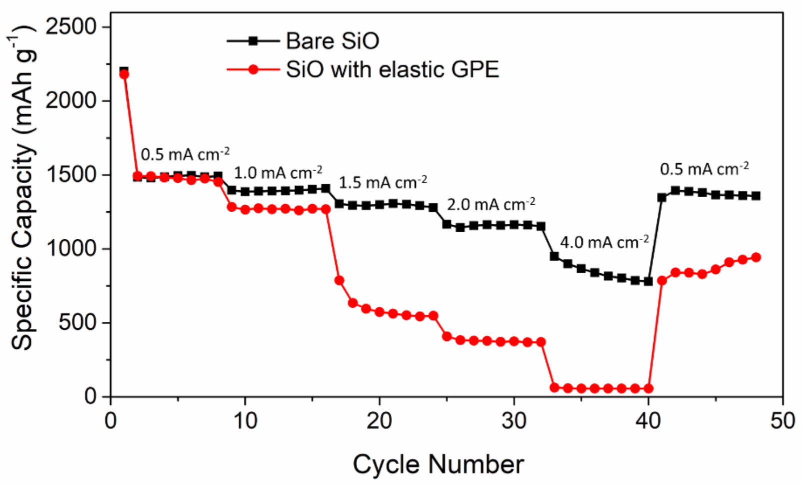

Supplementary Figure 15. Rate performance of the SiO electrodes with and without the copolymer 1 elastic GPE. The mass loading of SiO electrodes is 3.7 mg cm^{-2} and the polymer coating amount is 0.4 mg cm^{-2}.

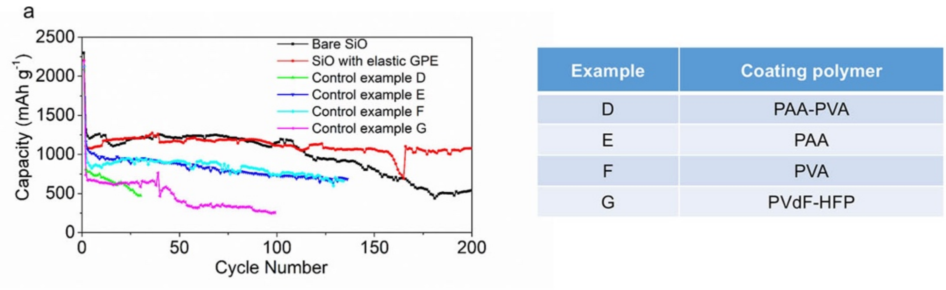

Example	Coating polymer
D	PAA-PVA
E	PAA
F	PVA
G	PVdF-HFP

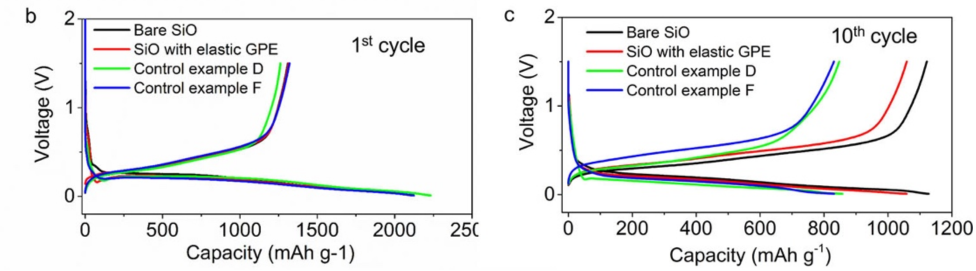

Supplementary Figure 16. Cycling stability comparison of the SiO anodes with the copolymer 1 and other polymers as the cushion. (**a**) Discharge capacities of the SiO electrodes. The control examples D, E, F and G contain PAA-PVA, PAA, PVA, and PVdF-HFP polymers, respectively, those used as the cushion. The polymer amount is 0.4 mg cm^{-2}. (**b**) The first-cycle voltage profiles. (**c**) The tenth-cycle voltage profiles.

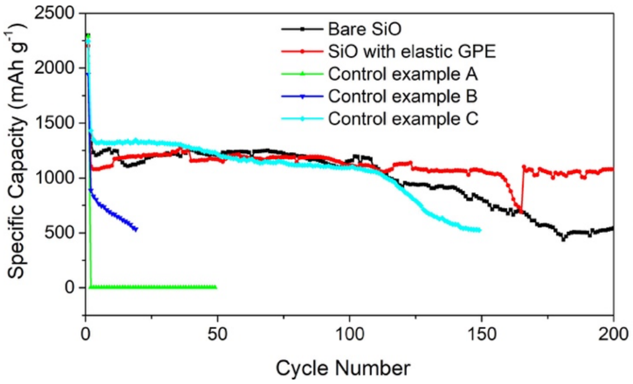

Example	Composition	Purpose of the example
A	SiO:SP:copolymer = 7:2:1	Copolymer used as an binder
B	SiO:SP:PAA-PVA:copolymer = 6:2:1:1	Copolymer used as an additional binder
C	SiO:SP:PAA-PVA = 6:2:2	Increased amount of the original binder

Supplementary Figure 17. Cycling stability of the SiO electrodes with different polymer binders. The bare SiO and SiO with the elastic GPE electrodes are composed of active SiO, conductive carbon and PAA-PVA binder in a mass ratio of 7:2:1. The mass loading of SiO electrodes is 3.7 mg cm^{-2}. The amount of polymer coating is 0.4 mg cm^{-2}. The control example A is composed of SiO, conductive carbon and the elastic copolymer binder at a mass ratio of 7:2:1. The mass loading of SiO electrode is 3.7 mg cm^{-2}. The control example B is composed of SiO, conductive carbon, PAA-PVA binder, and the copolymer with a mass ratio of 6:2:1:1. The mass loading of SiO electrode is 4.2 mg cm^{-2}. The control example C is composed SiO, conductive carbon and PAA-PVA binder at a mass ratio of 6:2:2. The mass loading of the SiO electrodes is 4.2 mg cm^{-2}.

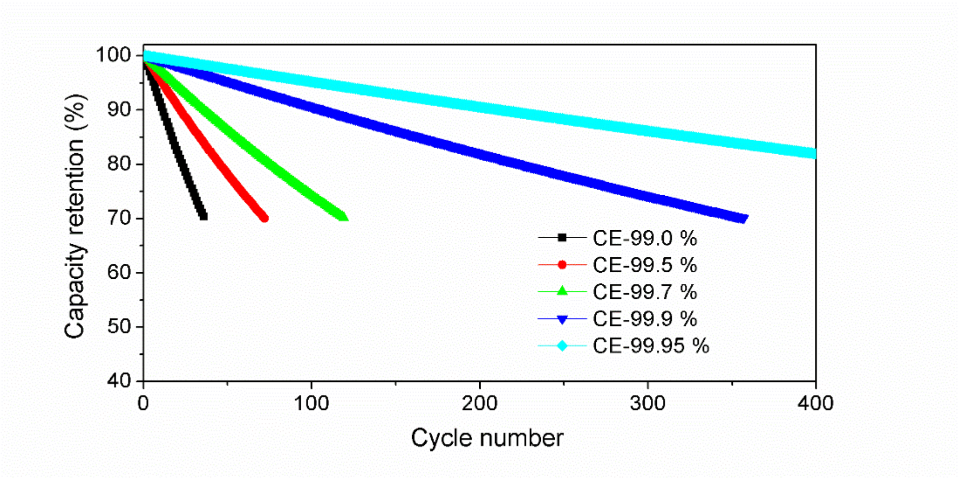

Supplementary Figure 18. The full-cell capacity retention as a function of Columbic efficiency and cycle number.

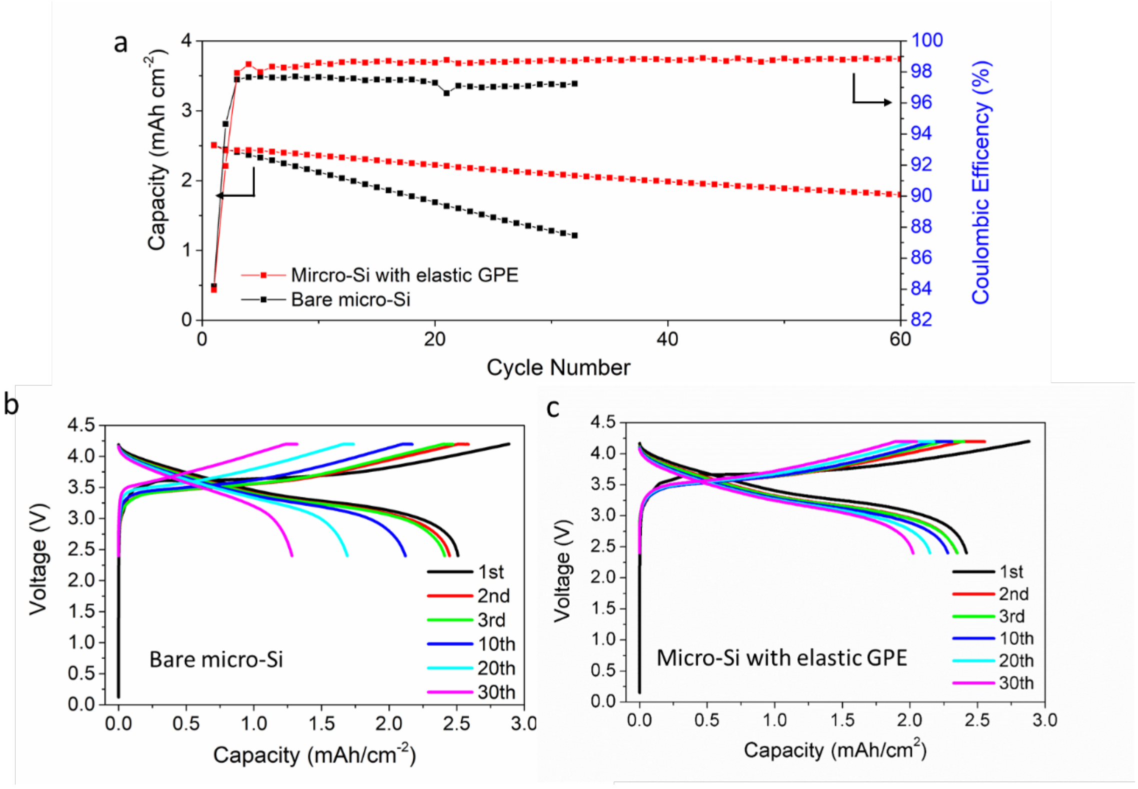

Supplementary Figure 19. Cycling stability of the micro-Si anodes (Si microparticle (1-3 µm)) with the elastic GPE. **(a)** Full-cell discharge capacities of micro-Si|NCM cells at a current density of 0.4 mA cm^{-2}, along with the Coulombic efficiency of the cell incorporating with the elastic GPE. (**b** and **c**) Corresponding voltage profiles of the cells without (b) and with (c) the elastic GPE. The mass loading of the micro-Si electrodes is 2.8 mg cm^{-2} and was directly used for cell assembling.

Supplemental Note 1. SEM characterization of pristine SiO electrodes with different amounts of the copolymer 1 coating.

We used a dilute copolymer 1 solution (5 w% in dimethylacetamide) to conduct the polymer coating. The polymer can be intentionally designed to fill in the pores inside SiO electrode. When the polymer amount is 0.4 mg cm^{-2}, the SiO electrode had a rough surface and we could distinguish SiO particles from conductive carbons (Supplemental Fig. 5a and 5b). The polymer is mainly in the pores rather than on the surface. Moreover, as a control sample, we increased the polymer amount to 0.7 mg cm^{-2}. The SiO electrode had a polymer coating layer on the surface (Supplemental Fig. 5c and 5d).

Supplemental Note 2. High resolution XPS spectra of pristine SiO electrodes.

In Supplemental Fig. 7, the copolymer 1 has characteristic O=C-N structure (peaks at 399.7 eV in the N 1s spectrum in Supplemental Fig. 7b), while bare SiO electrode shows no N 2s signal (Supplemental Fig. 7e). For the SiO electrode with the copolymer 1, characteristic O=C-N signals was observed in Supplemental Fig. 7h, which indicates that top surface of electrode is coated with copolymer 1. Meanwhile, the bare SiO electrode shows characteristic Si 2p peaks (Supplemental Fig. 7f) that comes from SiO, while the Si 2p peaks disappeared for the electrode with the copolymer 1 (Supplemental Fig. 7i). It shows the same results with N 1s peaks.

The C 1s peak in the spectrum of the SiO electrode with the copolymer 1 (Supplemental Fig. 7g) shows typical C-C bonds at 284.6 eV and C-O bonds at 286.0 eV, which is similar to that of the copolymer 1 (Supplemental Fig. 7a). In comparison, the C 1s peak for the bare SiO electrode (Supplemental Fig. 7d) shows not only the existence of C-C (284.6 eV) and C-O (286.0 eV) bonds, but also ester group (-COO-) at the peaks at 288.2 eV and 288.8 eV, which are coming from the conventional polyacrylic acid-polyvinyl alcohol (PAA-PVA) binder.

Supplemental Note 3. Electrodes stability test in liquid electrolyte under magnetic stirring.

We stirred the SiO electrodes vigorously in the electrolyte (1 M $LiPF_6$ in EC/DEC (1:1 v/v)). A large amount of the electrode materials was peeled off from the bare SiO electrode (Supplemental Fig. 9a and 9c), while the structure of the GPE-incorporated SiO electrode was maintained (Supplemental Fig. 9b and 9d).

Supplemental Note 4. Electrochemical performance of SiO electrode with different amounts of the copolymer 1.

When copolymer amount was 0.7 mg cm^{-2}, the specific capacity of the SiO anode was lower and faded rapidly, compared to that of 0.4 mg cm^{-2} (Supplemental Fig 14a). The excessive copolymer also induced large polarization of the cell (Supplemental Fig 14b and 14c).

Supplemental Note 5. Rate performance of the SiO electrodes with and without copolymer 1 elastic GPE.

As shown in Supplemental Fig. 15, the SiO electrode with elastic GPE has high reversible capacities when the current density is below 1.0 mA cm^{-2}, corresponding to a current rate of 0.4 C. The specific capacity started to drop when further increased the current density.

Supplemental Note 6. Cycling stability comparison of the SiO anodes with the copolymer 1 and other polymers as the cushion.

In addition, we studied other types of polymer as the cushion for SiO electrodes, including PAA-PVA (control example D), PAA (control example E), PVA (control example F), and PVdF-HFP (control example G). However, the SiO electrodes had low capacities and rapid capacity fading compared to the SiO electrode with the elastic GPE. The preparation of the electrodes followed the same approach. A current density of 1.0 mA cm^{-2} was used for battery cycling.

Supplemental Note 7. Cycling stability of the SiO electrodes with different polymer binders.

Firstly, we directly utilized the copolymer as binder to make SiO electrode (control example A). The SiO electrode has normal discharge capacity for 1st cycle, however it dropped below 10 mAh g^{-1} for the following cycles (Supplemental Fig. 17). The copolymer cannot bind SiO particles tightly and accommodate large volume change upon cycling.

Secondly, we mixed the copolymer with PAA-PVA binder and used the mixed polymer solution to make a slurry (control example B). The slurry was composed of SiO, conductive carbon, PAA-PVA binder, and the copolymer with a mass ratio of 6:2:1:1. The control example A has the same polymer composition as the SiO electrode with elastic GPE. In Supplemental Fig. 17, the SiO electrode with mixed polymer binder shows lower capacity and faster capacity fading than SiO electrode with elastic GPE.

Thirdly, we further increased the PAA-PVA binder content from 10 w% to 20 w% (control example C). The control example C shows similar cycling stability as bare SiO electrode with 10 w% polymer binder. The SiO electrode has high specific capacity of 1300 mAh g^{-1} for 40 cycles, which is higher than bare SiO electrode with 10 w% of polymer binder. With more polymer binder, the SiO particles can tolerate larger volume change in each cycle, thus showing larger capacity. However, the capacity starts to fades after 40 cycles and it drops quickly after 120 cycles.

Supplemental Note 8. The full-cell capacity retention as a function of Columbic efficiency and cycle number.

Supposed: the initial capacity is fixed to unit; the Columbic efficiency is defined as C(N+1)/C (N), where as the C(N) is the cell capacity at Nth cycle; the CE are the same at different cycles.

$C(2) = C(1)*CE$

$C(3) = C(2)*CE = C(1)*CE^2$

$C(4) = C(3)*CE = C(1)*CE^3$

……

$C(N) = C(N-1)*CE = C(1)*CE^N$

If the CE is 99.0%, the capacity retention drops below 80% after 24 cycles.

If the CE is 99.5%, the capacity retention drops below 80% after 46 cycles.

If the CE is 99.7%, the capacity retention drops below 80% after 76 cycles.

If the CE is 99.9%, the capacity retention drops below 80% after 225 cycles.

If the CE is 99.95%, the capacity retention drops below 80% after 448 cycles.

Supplemental Note 9. Cycling stability of the micro-Si anodes (Si microparticle (1-3 µm)) with the elastic GPE.

As a control experiment, we prepared Si microparticle electrodes following the same procedure. Then we paired micro-Si electrodes with NCM cathode with a mass loading of 15 mg cm^{-2}. For bare micro-SiO electrode, the capacity retention was 50% after 30 cycles, which indicated a full cell Coulombic efficiency of 97.7%. For micro-Si electrode with polymer coating, the full cell exhibited an initial areal capacity of 2.50 mAh cm^{-2} and it dropped to 1.80 mAh cm^{-2} after 60 cycles (Supplemental Fig. 19). The capacity retention was 72% after 60 cycles, which indicated a full cell Coulombic efficiency of 99.5%. Thus the copolymer coating on micro-Si electrode also improved full cell cycling stability.
